# Spatial multiomic profiling reveals the novel polarization of foamy macrophages within necrotic granulomatous lesions developed in lungs of C3HeB/FeJ mice infected with *Mycobacterium tuberculosis*

**DOI:** 10.3389/fcimb.2022.968543

**Published:** 2022-09-27

**Authors:** Shintaro Seto, Hajime Nakamura, Tz-Chun Guo, Haruka Hikichi, Keiko Wakabayashi, Akiko Miyabayashi, Toshi Nagata, Minako Hijikata, Naoto Keicho

**Affiliations:** ^1^ Department of Pathophysiology and Host Defense, The Research Institute of Tuberculosis, Japan Anti-Tuberculosis Association, Tokyo, Japan; ^2^ Department of Health Science, Hamamatsu University School of Medicine, Hamamatsu, Japan; ^3^ Vice Director, The Research Institute of Tuberculosis, Japan Anti-Tuberculosis Association, Tokyo, Japan

**Keywords:** Tuberculosis, *Mycobacterium tuberculosis*, C3HeB/FeJ, necrotic granuloma, foamy macrophage, proteomics, transcriptomics

## Abstract

Infection with *Mycobacterium tuberculosis* leads to the development of tuberculosis (TB) with the formation of granulomatous lesions. Foamy macrophages (FM) are a hallmark of TB granulomas, because they provide the primary platform of *M. tuberculosis* proliferation and the main source of caseous necrosis. In this study, we applied spatial multiomic profiling to identify the signatures of FM within the necrotic granulomas developed in a mouse model resembling human TB histopathology. C3HeB/FeJ mice were infected with *M. tuberculosis* to induce the formation of necrotic granulomas in the lungs. Using laser microdissection, necrotic granulomas were fractionated into three distinct regions, including the central caseous necrosis, the rim containing FM, and the peripheral layer of macrophages and lymphocytes, and subjected to proteomic and transcriptomic analyses. Comparison of proteomic and transcriptomic analyses of three distinct granulomatous regions revealed that four proteins/genes are commonly enriched in the rim region. Immunohistochemistry confirmed the localization of identified signatures to the rim of necrotic granulomas. We also investigated the localization of the representative markers for M1 macrophages in granulomas because the signatures of the rim included M2 macrophage markers. The localization of both macrophage markers suggests that FM in necrotic granulomas possessed the features of M1 or M2 macrophages. Gene set enrichment analysis of transcriptomic profiling revealed the upregulation of genes related to M2 macrophage activation and mTORC1 signaling in the rim. These results will provide new insights into the process of FM biogenesis, leading to further understanding of the pathophysiology of TB granulomas.

## Introduction

Tuberculosis (TB) is a chronic inflammatory disease caused by *Mycobacterium tuberculosis* infection, with high morbidity and mortality worldwide. A total of 1.5 million people died from TB in 2020, increasing from 1.4 million in 2019, due to reduced access to diagnosis and treatment (WHO, 2021). In the process of infection, *M. tuberculosis* bacilli are inhaled into the alveoli *via* the respiratory tract and subsequently phagocytosed by alveolar macrophages. Phagocytosed bacilli avoid host defense mechanisms by phagosome maturation arrest, the inhibition of phagolysosome biogenesis and protection from reactive oxidative radicals (Weiss and Schaible, 2015). Infected macrophages induce inflammatory responses, resulting in the recruitment of uninfected macrophages and the formation of cell aggregates, which are known as granulomas. The translocation of *M. tuberculosis* from phagosomes to cytosol in infected macrophages also leads to the production of inflammatory cytokines and host cell death (Groschel et al., 2016). Upon the establishment of adaptive immunity, lymphocytes surround the macrophage-rich granulomatous lesions to contain the infected bacilli (Ramakrishnan, 2012; Pagan and Ramakrishnan, 2014).

During the formation of TB granulomas, *M. tuberculosis*-infected macrophages accumulate lipid droplets (LD) within their cytoplasm and differentiate into foamy macrophages (FM) (Peyron et al., 2008). LD within FM are thought to serve as a source of nutrients for the intracellular bacilli, thus providing a preferable environment for the bacterium (Peyron et al., 2008; Singh et al., 2012). Accumulation of LD is shown to modulate the immune response of FM, leading to the production of inflammatory cytokines and eicosanoids, suggesting that FM influence the outcome of *M. tuberculosis* infection (Agarwal et al., 2021). With the progression of the disease, FM play a central role in granuloma development, maintenance, and infection dissemination (Russell et al., 2009). In the progression of granuloma formation, *M. tuberculosis*-infected FM fall into necrosis, resulting in the formation of a necrotic core during the development of the necrotic granuloma. Inside the necrotic core, the tissue environment is altered to further lipid-rich and hypoxic conditions, leading to growth inhibition and a shift to the dormant state of *M. tuberculosis* (Sarathy and Dartois, 2020). In the dissemination process, *M. tuberculosis*-infected FM are released from the original granulomas, followed by the formation of secondary granulomatous lesions (Russell et al., 2009).

Mouse models provide important tools for investigating TB immunopathology and for developing drugs and vaccines against TB (Cooper, 2014). However, the two most available inbred laboratory mouse strains, C57BL/6 and BALB/c, have not been demonstrated to generate necrotic granulomatous lesions upon *M. tuberculosis* infection (Rhoades et al., 1997; Orme, 2003). Kramnik et al. originally reported the development of necrotic granulomas within the lungs of C3HeB/FeJ mice infected with *M. tuberculosis* (Kramnik et al., 1998), followed by further reports assessing their histopathology and pathogenicity in detail (Driver et al., 2012; Harper et al., 2012; Irwin et al., 2015; Lanoix et al., 2015). Blood transcriptomic profiling and the ultralow-dose aerosol infection model also suggest that this mouse strain infected with *M. tuberculosis* represents the relevant model of active TB disease (Moreira-Teixeira et al., 2020; Plumlee et al., 2021). Proliferating *M. tuberculosis* bacilli were shown to be enriched in the rim of necrotic granulomas where large numbers of FM have accumulated in this mouse model (Walter et al., 2021), suggesting that FM is a major platform for *M. tuberculosis* proliferation in necrotic granulomas in C3HeB/FeJ mice.

Recent reports of genome-wide expression profiling have demonstrated the detailed features of *M. tuberculosis*-infected macrophages and the implication of lipid metabolism in *M. tuberculosis* proliferation (Huang et al., 2018; Russell et al., 2019; Pisu et al., 2020; Pisu et al., 2021). In addition, several intracellular signaling pathways including mTORC1 signaling have been shown to regulate LD formation in *M. tuberculosis*-infected macrophages (Mahajan et al., 2012; Guerrini et al., 2018; Knight et al., 2018; Brandenburg et al., 2021). However, the protein and gene expression profiles of FM within granulomas are not fully understood. In this study, we evaluated the expression profiles of proteins and genes enriched in the rim of necrotic granulomatous lesions using a mouse model relevant to active TB disease. Spatial multiomic profiling and the localization of genes identified in the signatures suggest the presence of M2 macrophages and FM derived from M2 macrophages within the rim of necrotic granulomas as well as M1 macrophages. The results of this study propose that M2 macrophage-derived FM or mTORC1 signaling pathway in the rim region of necrotic granulomas are potential targets for novel TB diagnostic or host-directed therapeutic drugs.

## Results

### Development of necrotic granulomatous lesions in the lungs of C3HeB/FeJ mice infected with *M. tuberculosis*

**Figure 1:**
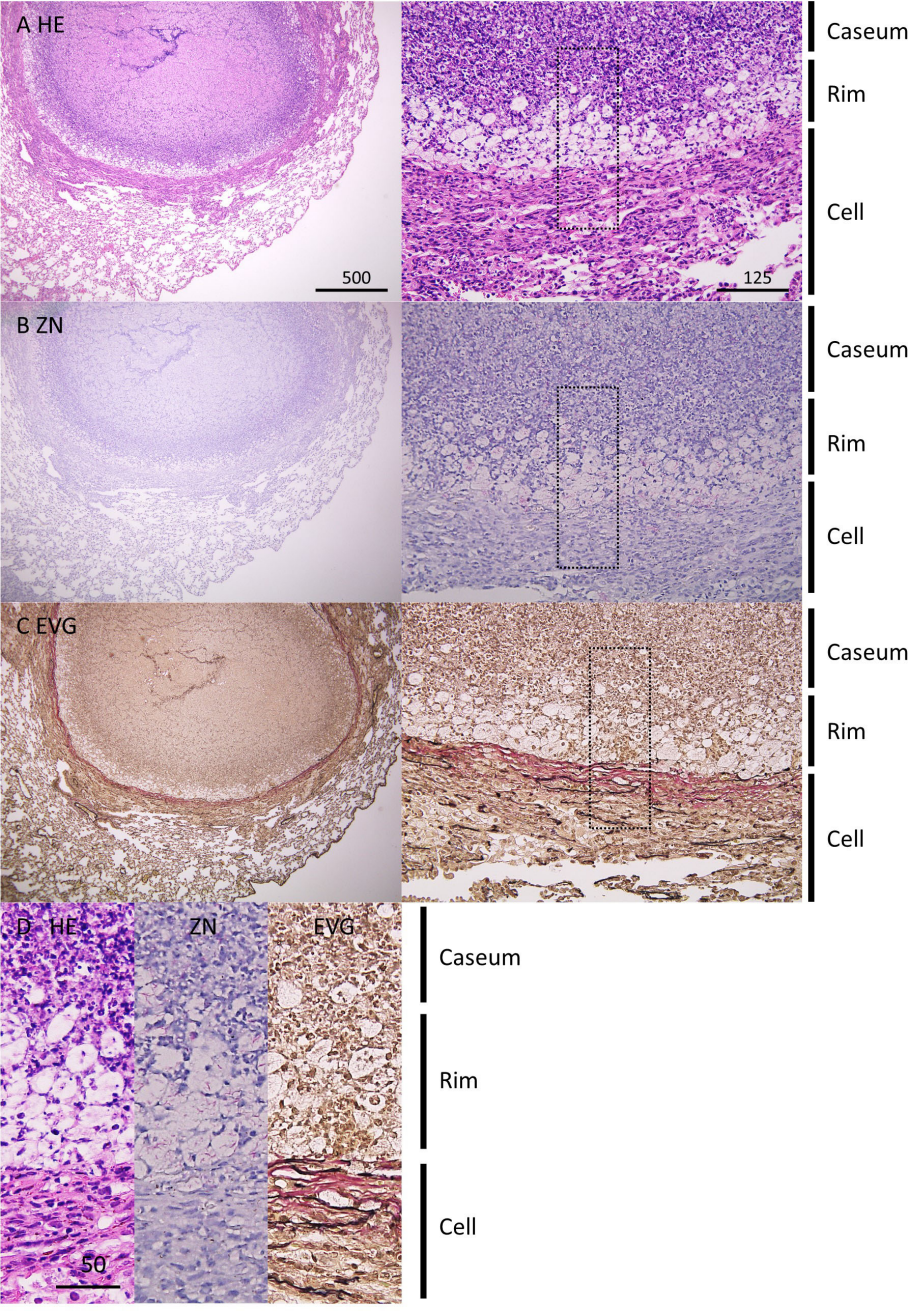
Pathological features of necrotic granulomatous lesions developed in lungs of *M. tuberculosis*-infected C3HeB/FeJ mice. **(A–C)** Infected lungs with necrotic granulomatous lesions at 8 weeks p.i. were stained with hematoxylin-eosin (HE) **(A)** and Ziehl-Neelsen (ZN) staining **(B)**. Elastica van Gieson (EVG) staining was carried out to visualize collagen (pink) and elastic (black) fibers **(C)**. The right panels are enlarged images of the left panels. **(D)** Comparison of images with HE, ZN, and EVG staining of necrotic granulomas. The regions enclosed by the dotted lines in **(A–C)** were enlarged. The regions corresponding to necrotic caseum (Caseum), rim enriched foamy macrophages (Rim), and peripheral macrophages and lymphocytes (Cell) are indicated.

In this study, we infected C3HeB/FeJ mice with *M. tuberculosis* Erdman strain at 500 CFU *via* the intranasal route. At 8 weeks postinfection (p.i.), three distinct lesion types were observed in infected lungs as reported previously (Driver et al., 2012; Harper et al., 2012; Irwin et al., 2015). Type I lesions, which resembled typical human necrotic granulomas, comprised caseous necrosis (Caseum) in the center that was surrounded by epithelioid macrophages and lymphocytes (Cell) (**Figure 1A**). Between the central Caseum and the peripheral Cell, a rim (Rim) enriched with vacuolated cells, a characteristic of FM, was observed. Ziehl-Neelsen (ZN) staining demonstrated that a large number of acid-fast bacilli (AFB) localized in Rim and Caseum (**Figure 1B**). Elastica van Gieson (EVG) staining revealed that a boundary containing collagen fibers separated Rim and Cell regions (**Figure 1C**), as previously reported (Irwin et al., 2015). High magnification images of HE and EVG staining showed that Rim was composed of a layer of FM between a dense neutrophil layer in Caseum and the collagen and elastic fibers in Cell (**Figure 1D**), as reported previously (Irwin et al., 2015; Boute et al., 2017). ZN staining showed that the vacuolated cells in Rim accumulated intracellular bacilli, while extracellular bacilli localized in Caseum. Five out of 13 infected mice developed type I lesions in the lungs. Three mice exhibited mainly type II lesions characterized by ill-defined consolidation with a massive number of AFB covering a large area of the lungs. The remaining mice displayed only type III lesions, consisting of aggregates of macrophages and lymphocyte clusters in infected lungs. These results suggest that intranasal infection of mice with *M. tuberculosis* successfully promoted the development of granulomatous lesions including necrotic granulomas in C3HeB/FeJ mice.

### Spatial multiomic profiling of necrotic granulomas

We conducted spatial proteomic and transcriptomic analyses to identify the signatures of the expression of proteins and genes in FM within Rim region of necrotic granulomas. We microdissected necrotic granulomas of hematoxylin-stained formalin-fixed, paraffin-embedded (FFPE) samples into three regions containing Caseum, Rim, and Cell separately according to the diagnosis performed by comparing samples stained with hematoxylin and eosin (HE) and EVG as previously described (Seto et al., 2020). Proteins or RNA were extracted from these regions and subjected to further analyses.

**Figure 2:**
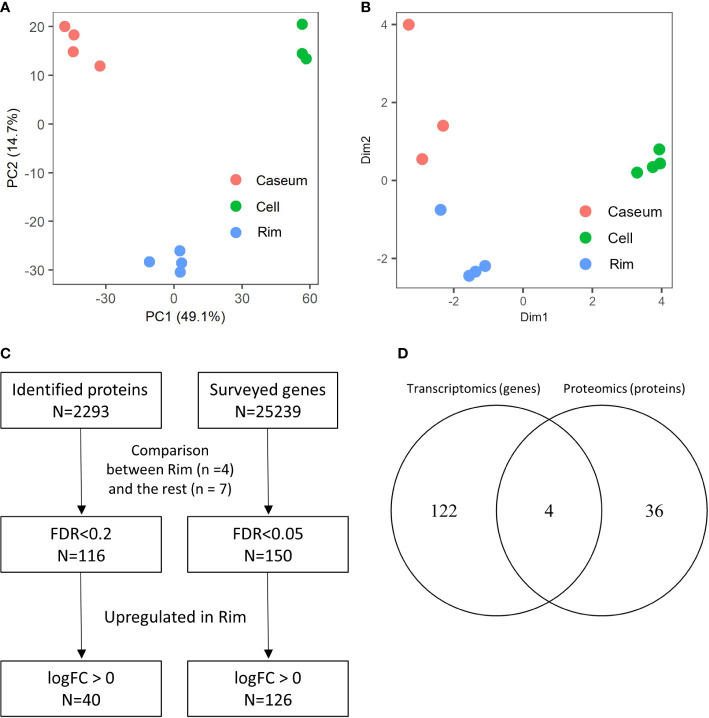
Spatial proteomic and transcriptomic profiling to determine signatures enriched in Rim region within necrotic granulomas. Necrotic granulomas at 8 weeks p.i. were microdissected into three regions containing Caseum, Rim, and Cell. Proteins and RNA were extracted from granulomatous regions, followed by proteomic and transcriptomic analyses, respectively. **(A)** Principal component analysis (PCA) of granulomatous regions based on label-free quantification (LFQ) values of proteomic analysis. The first two components, PC1 and PC2, accounted for 49.1% and 14.7% of the variability from all data, respectively, and are shown in the plot. **(B)** Multi-dimensional scaling (MDS) plot of distances between gene expression profiles of granulomatous regions based on differential gene expression results obtained from transcriptomic profiling. **(C)** Flowchart describing the number of proteins and genes used to select signatures for Rim. The expression levels between four samples derived from Rim and seven samples derived from the other granulomatous regions were compared in proteomic and transcriptomic analyses. Welch’s *t* test with FDR correction, and generalized linear models and quasi-likelihood tests of edgeR were applied to identify the differentially expressed proteins and genes, respectively. **(D)** Venn diagram comparing the number of proteins or genes whose expression levels were upregulated in Rim region. The numbers of proteins or genes whose expression levels in Rim regions were significantly higher than those in the other two regions are shown.

Proteins extracted from the microdissected regions of necrotic granulomas were digested by trypsin and subsequently subjected to a tandem mass spectrometer coupled to liquid chromatography (LC–MS/MS). The mass spectrometry raw data were initially processed using MaxQuant software (Cox and Mann, 2008) against the UniProt FASTA mouse database. In total, 13419 peptides were identified and mapped to 2293 protein groups, followed by the application of the label-free quantification (LFQ) algorithm to compare the abundances of proteins in the samples (**Supplementary Table S1**) (Cox et al., 2014). Principal component analysis based on LFQ values of proteins demonstrated sufficient discrimination of protein expression profiles in these granulomatous regions (**Figure 2A**). We next assessed gene expression profiles in necrotic granulomas by RNA sequencing (RNA-seq). RNA was extracted from microdissected regions, followed by the construction of cDNA libraries and RNA-seq analysis (**Supplementary Table S2**). Multi-dimensional scaling also clearly separated the gene expression profiles of the granulomatous regions (**Figure 2B**). These results indicate that necrotic granulomas were fractionated into three distinct regions where the expression profiles of proteins or RNA were different.

To identify proteins and genes whose expression levels were significantly upregulated in Rim region, we compared the differentially expressed proteins and genes between Rim and the other regions within the necrotic granulomas (**Figure 2C**). Heatmaps based on hierarchical clustering analysis demonstrated that the expression levels of selected proteins and genes in Rim were different from those in other regions (**Supplementary Figure S1**). A Venn diagram demonstrated the identification of four proteins/genes commonly upregulated in Rim region (**Figure 2D**). Their characteristics are summarized in **Table 1**.

**Table 1:** Candidates of signatures for FM.

Gene	Gene name	Function in atherosclerosis and *M. tuberculosis* infection
*Plin2*	Perilipin 2	LD formation and foam cell differentiation in atherosclerosis (Paul et al., 2008). Localization to FM in TB necrotic granulomas (Kim et al., 2010).
*Msr1*	Macrophage scavenger receptor 1	Promotion of foam cell differentiation in atherosclerosis (Suzuki et al., 1997). Anti-inflammatory M2 macrophage marker in mice (Ruffell et al., 2009)
*Arg1*	Arginase 1	Anti-inflammatory M2 macrophage marker in mice (Ruffell et al., 2009)
*Lgals3*	Galectin 3	Protection against *M. tuberculosis* infection (Chauhan et al., 2016)

### Polarization of FM within necrotic granulomas

**Figure 3:**
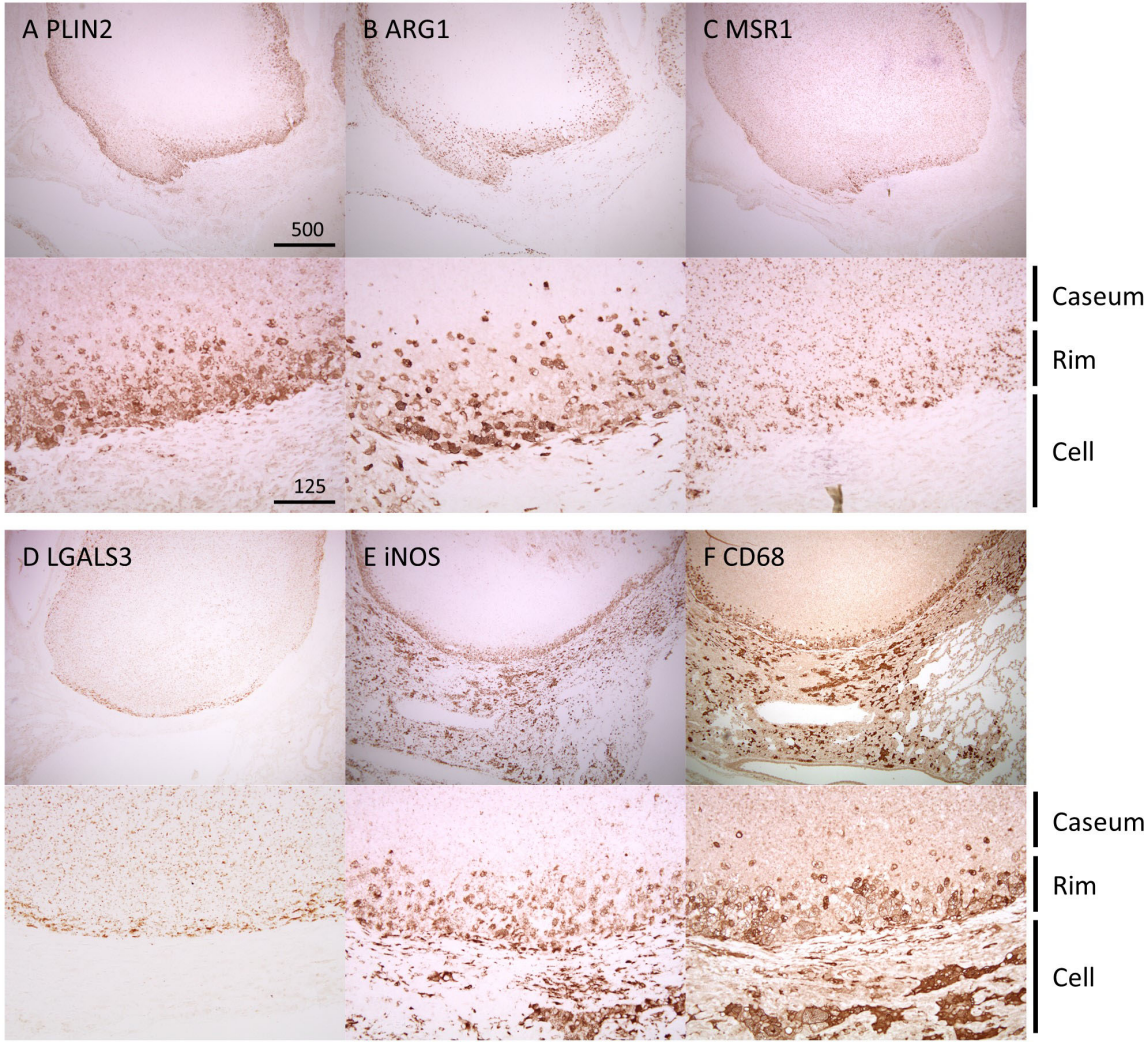
Localization of Rim signatures within necrotic granulomas. IHC was performed to stain necrotic granulomas at 8 weeks p.i. for Rim signatures of PLIN2 **(A)**, ARG1 **(B)**, MSR1 **(C)**, and LGALS3 **(D)**. IHC images for the M1 macrophage markers iNOS **(E)** and CD68 **(F)** are also shown. The lower panels are enlarged images of the upper panels. The regions corresponding to Caseum, Rim, and Cell of necrotic granulomas are indicated.

We performed immunohistochemistry (IHC) to observe the localization of the identified proteins within necrotic granulomas at 8 weeks p.i. (**Figure 3**). IHC for Perilipin-2 (PLIN2), Arginase-1 (ARG1), Macrophage scavenger receptor 1 (MSR1), and Galectin-3 (LGALS3) showed strong signals in the cells corresponding to Rim region in necrotic granulomas (**Figure 3**). The signals of these proteins corresponded to distinct subcellular localization. PLIN2 and ARG1 localized to the cytosol, and MSR1 and LGALS3 localized to the plasma membrane. PLIN2^+^ or ARG1^+^ cells exhibited vacuolation. Considering that ARG1 and MSR1 are markers for M2 macrophages (Ruffell et al., 2009), we investigated the localization of the markers for M1 macrophages, including inducible nitric oxide synthase (iNOS) and CD68 (**Figure 3**). Both iNOS and CD68 localized to Rim and Cell regions in necrotic granulomas as reported previously (Carow et al., 2019).

**Figure 4:**
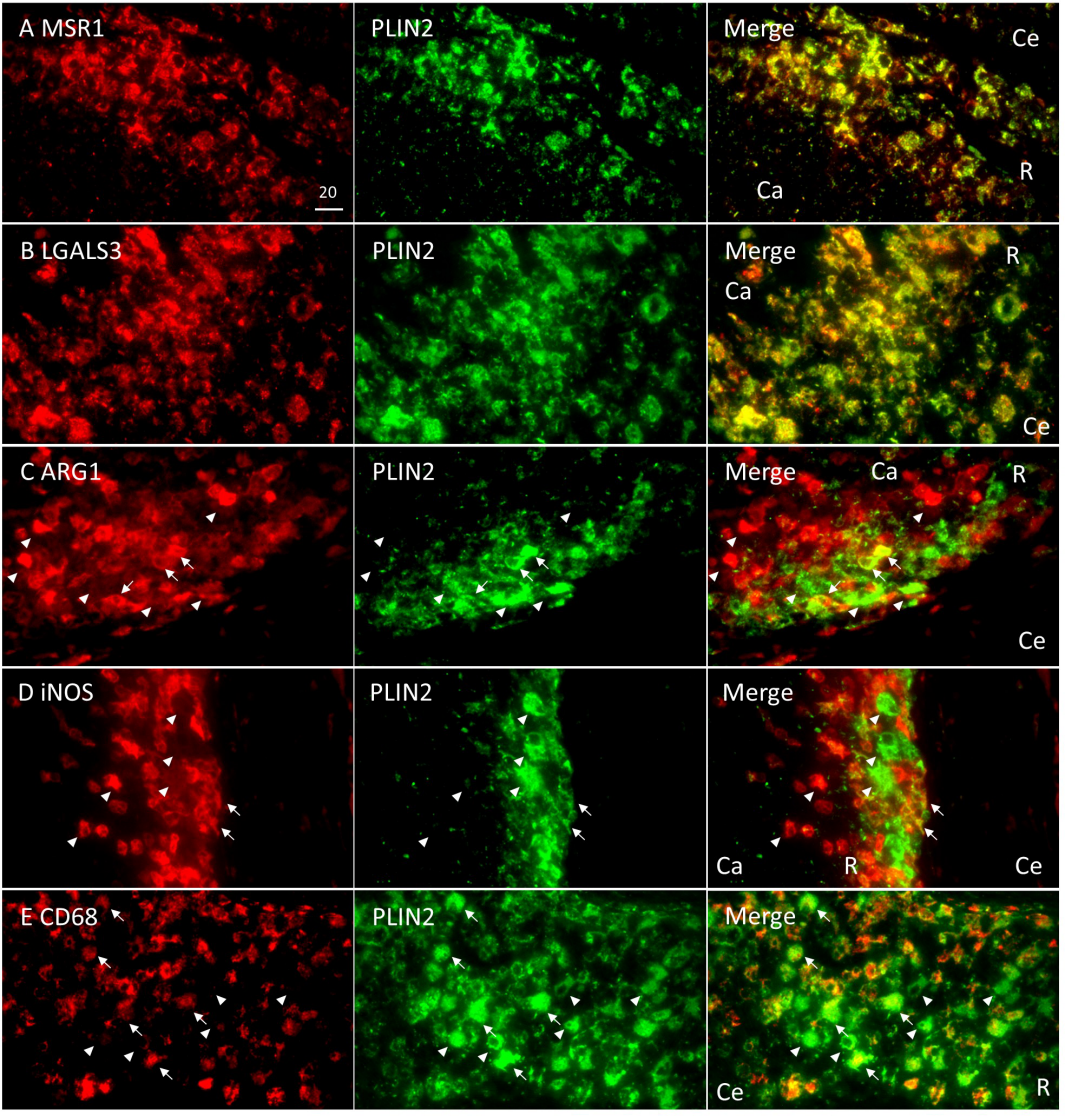
Colocalization of Rim signatures and macrophage polarization markers with PLIN2 in Rim region of necrotic granulomas. Immunofluorescence microscopy (IFM) was performed to stain necrotic granulomas at 8 weeks p.i. with rabbit anti-MSR1 and guinea pig (GP) anti-PLIN2 **(A)**, rabbit anti-LGALS3 and GP anti-PLIN2 **(B)** rabbit anti-ARG1 and GP anti-PLIN2 **(C)**, rabbit anti-iNOS and GP anti-PLIN2 **(D)**, and rabbit anti-CD68 and GP anti-PLIN2 **(E)** antibodies. Arrows and arrowheads indicate both marker and PLIN2-positive cells and either marker or PLIN2 alone-positive cells, respectively. The regions corresponding to Caseum (Ca), Rim (R), and Cell (Ce) of necrotic granulomas are indicated in merged images.

**Figure 5:**
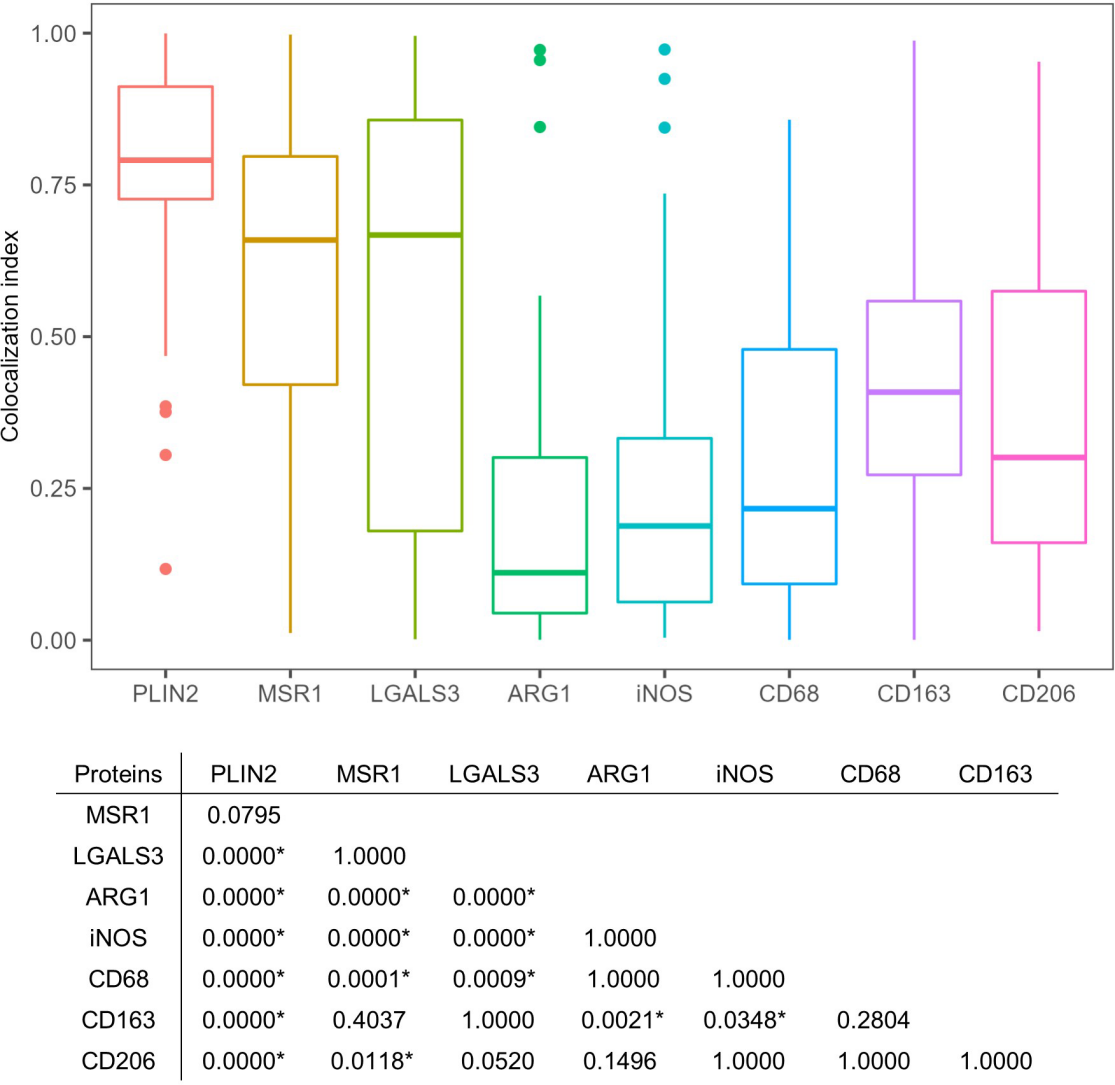
Quantitative colocalization of signatures with PLIN2^+^ cells in necrotic granulomas. The colocalization index was estimated by dividing the area of the indicated marker protein colocalizing with PLIN2 by that of PLIN2 in the image. The colocalization index was calculated from approximately 50 images of cells stained with antibodies against PLIN2 and PLIN2 (PLIN2), MSR1 and PLIN2 (MSR1), LGALS3 and PLIN2 (LGALS3), ARG1 and PLIN2 (ARG1), iNOS and PLIN2 (iNOS), CD68 and PLIN2 (CD68), CD163 and PLIN2 (CD163), and CD206 and PLIN2 (CD206). The results of Dunn’s multiple-comparison test between the colocalization indices of the indicated proteins to PLIN2 are also shown. Asterisks indicate significant differences (*P* < 0.05).

Next, we surveyed the colocalization of these markers with PLIN2 by immunofluorescence microscopy (IFM) (**Figure 4**). PLIN2 has been reported to promote LD formation and form cell differentiation in atherosclerosis (Paul et al., 2008), and its subcellular localization is associated with LD (Itabe et al., 2017). Furthermore, PLIN2 is localized to FM in human necrotic granulomas (Kim et al., 2010). Therefore, we defined PLIN2^+^ cells within necrotic granulomas as FM. Localization of MSR1 and LGALS3 was consistent with that of PLIN2, indicating that these proteins are also markers for FM within necrotic granulomas. These results also suggest that PLIN2^+^ cells in granulomas are derived from macrophages because *Msr1* and *Lgals3* are primarily expressed by monocytes/macrophages (**Supplementary Figure S2A**). Other macrophage activation markers, including ARG1, iNOS, and CD68, did not localize to some proportion of PLIN2^+^ cells, and vice versa (**Figure 4**). Quantitative colocalization analysis also revealed that MSR1 colocalized with PLIN2^+^ cells, while the other M2 marker, ARG1 and the M1 markers iNOS and CD68 showed colocalization with some proportion of PLIN2^+^ cells (**Figure 5**). We further investigated the localization of CD163 and CD206, other markers of M2 macrophages within necrotic granulomas, resulting in their localization pattern similar to that of MSR1 and LGALS3 (**Supplementary Figure S3** and **Figure 5**). We also found that 40-60% of CD68^+^, CD163^+^, or CD206^+^ macrophages were differentiated into FM by estimating the PLIN2^+^ cells in the marker-positive cells (**Supplementary Figure S2B**). In conclusion, these results suggest that MSR1 is the marker for FM rather than for M2 macrophage in necrotic granulomas of C3HeB/FeJ mice, and it is conceivable that polarized FM are differentiated from FM or polarized macrophages are further differentiated into FM within Rim.

**Figure 6:**
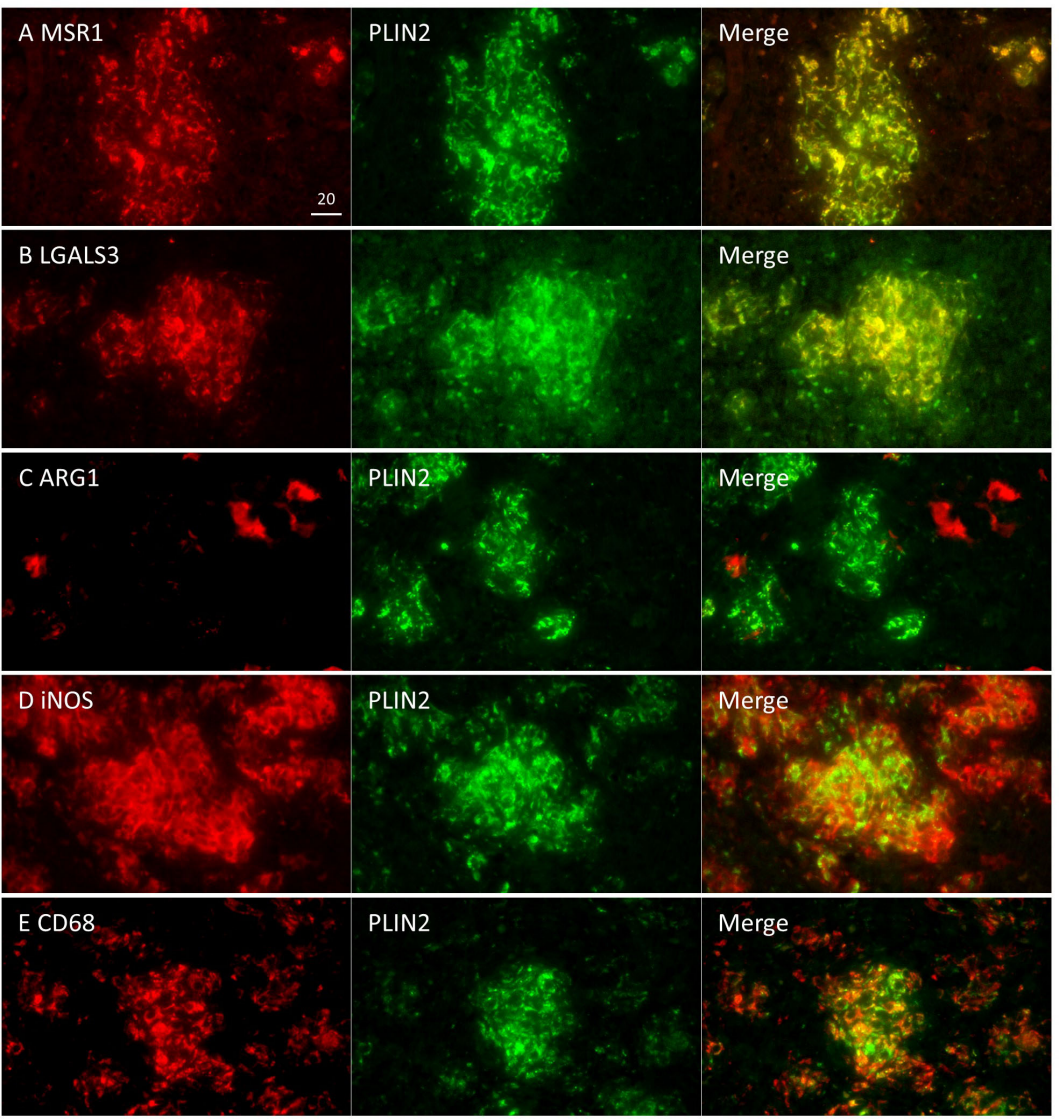
Localization of FM markers and macrophage activation markers in non-necrotic granulomas. Localization of MSR1 **(A)**, LGALS3 **(B)**, iNOS **(C)**, CD68 **(D)**, and ARG1 **(E)** to PLIN2^+^ cells was examined in non-necrotic granulomas at 8 weeks p.i.

In non-necrotic granulomas at 8 weeks p.i., localization of MSR1 or LGALS3 was consistent with that of PLIN2 (**Figure 6**), suggesting that these two proteins are also the marker for FM in non-necrotic granulomas. Colocalization of iNOS or CD68 with PLIN2 was also observed, while ARG1 was not colocalized with PLIN2, suggesting that FM within non-necrotic granulomas drive their activation to M1 macrophages. Moreover, the proportion of ARG1^+^ cells in non-necrotic granulomas was smaller than that in Rim of necrotic granulomas. Taken together, these results suggest that MSR1 and LGALS3 function as signatures of FM, which are polarized to M1 macrophages in non-necrotic granulomas developed in C3HeB/FeJ mice.

### Activation of M2 macrophage and mTORC1 signaling in Rim region within necrotic granulomas

**Figure 7:**
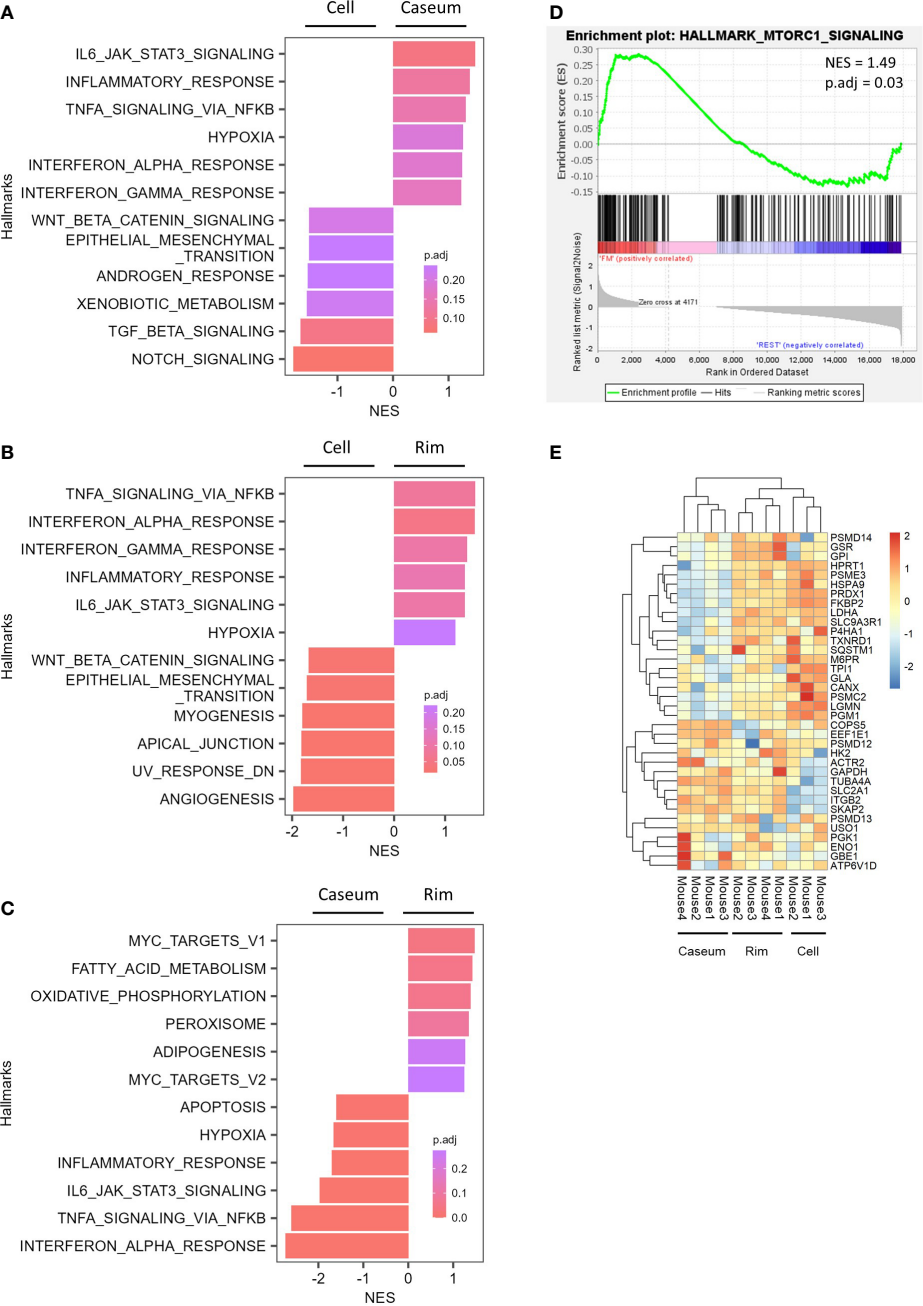
Expression of genes related to the activation of M2 macrophage and mTORC1 signaling in Rim of necrotic granulomas. **(A–C)** Gene set enrichment analysis (GSEA) showing enriched gene sets comparing between Caseum and Cell **(B)**, Rim and Caseum **(C)**, and Rim and Cell **(D)** fractions. GSEA was carried out using hallmark gene sets from Molecular Signatures Database. Normalized enrichment scores (NES) of the top six ranked hallmark gene sets are plotted with the color representing the adjusted *P* value (p.adj). **(D)** Enrichment plot for genes related to mTORC1 signaling. GSEA was carried out between Rim and other fractions. NES and p.adj are also shown. **(E)** Heatmap of hierarchical clustering of proteins related to fatty acid metabolism, oxidative phosphorylation, and mTORC1 signaling which correspond to the genes listed as the core enrichment in GSEA. Hierarchical clustering analysis was carried out based on the LFQ values of the indicated proteins.

We investigated the expression features of genes among necrotic granulomatous regions by gene set enrichment analysis (GSEA). Comparing gene expression profiles between Rim and Cell revealed similar trends to those between Caseum and Cell; the hallmark genes of WNT-β catenin signaling and epithelial mesenchymal transition signaling pathways were activated in Cell region, while those of inflammatory and immune pathways including type I and type II responses were enriched in Caseum or Rim regions (**Figures 7A, B**). Moreover, the expression of genes related to hypoxia was also upregulated in Caseum and Rim. These results reflect the condition of necrotic granulomas: inflammatory and hypoxic activity increased from Cell to Caseum, and cell proliferative activity was more potent from Caseum to Cell. Similar trends were observed in the proteomic analysis (**Supplementary Figure S4**).

In comparing the gene expression profile between Rim and Caseum, we found that genes related to adipogenesis were enriched in Rim (**Figure 7C**). Furthermore, the expression levels of hallmark genes of fatty acid metabolism and oxidative phosphorylation were upregulated in Rim region compared to Caseum. Gene enrichment analysis revealed that core enrichment genes in the hallmark of fatty acid metabolism included genes related to fatty acid oxidation (**Supplementary Figure S5** and **Table S3**). The activation of these metabolic pathways implying the polarization of M2 macrophages (Batista-Gonzalez et al., 2019) suggests that M2 macrophages were activated in Rim compared to Caseum. We subsequently compared the gene expression profiles between Rim and other regions by GSEA and found that the expression of genes related to mTORC1 signaling was upregulated in Rim region (**Figure 7D**). The expression of proteins in this pathway, which were listed as core enrichment in GSEA, was also upregulated in Rim compared to Caseum or Cell regions (**Figure 7E**). These results suggest that the activation of mTORC1 signaling pathway is elevated in Rim regions of necrotic granulomas.

**Figure 8:**
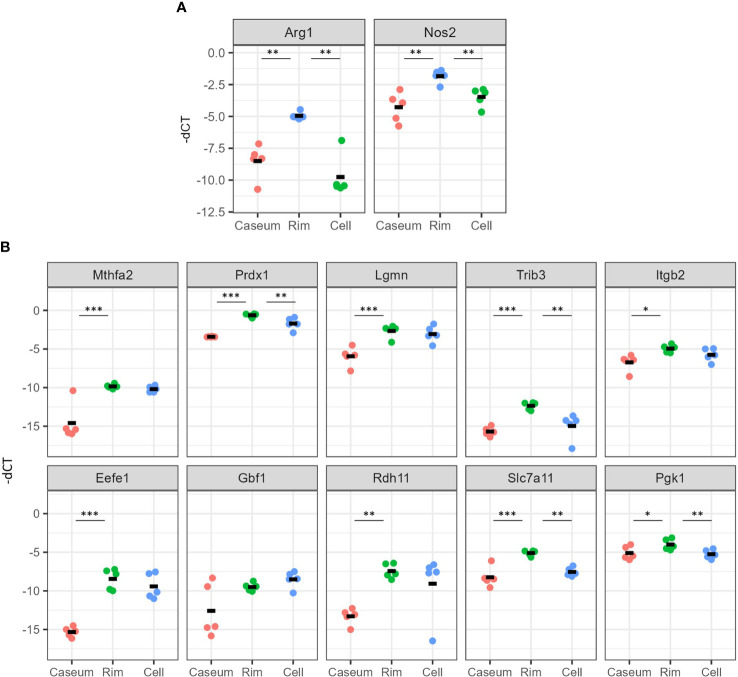
Verification of gene expression related to activation of M2 macrophages and mTORC1 signaling in necrotic granulomatous regions. RNA extracted from necrotic granulomatous regions (n = 5) was subjected to quantitative reverse-transcription PCR. Genes related to the macrophage activation markers, *Arg1* and *Nos2* **(A)** and mTORC1 signaling **(B)**, which are listed as the top 10 genes for the core enrichment in **Figure 6D**, were examined. The Ct values for the indicated genes normalized to that of *Actb* (-dCt) are indicated. *P < 0.05, **P < 0.01, ***P < 0.005 (Dunnett multiple comparison test following ANOVA).

We verified the gene expression results related to macrophage polarization and mTORC1 signaling by quantitative reverse transcription-PCR (qRT–PCR). We examined the gene expression of *Arg1* and *Nos2* and found that the expression levels of both genes were significantly higher in Rim region than in the other regions (**Figure 8A**). We surveyed the expression levels of the top ten genes of core enrichment in GSEA for mTORC1 signaling as indicated in **Figure 6C**, resulting in significantly higher levels of nine of these genes in Rim region than in Caseum, and the levels of four of these genes were higher in Rim region than in Cell region (**Figure 8B**). These results showed that M2 macrophages as well as M1 macrophages and mTORC1 signaling were activated in Rim region.

## Discussion

C3HeB/FeJ mice infected with *M. tuberculosis* are reported to develop necrotic granulomatous lesions in infected lungs (Kramnik et al., 1998; Driver et al., 2012; Harper et al., 2012; Irwin et al., 2015; Lanoix et al., 2015). The locus of *Super susceptibility to tuberculosis 1* (*Sst1*) containing *Sp110* and *Sp140* genes is involved in sensitivity to *M. tuberculosis* infection in C3eB/FeJ mice (Kramnik et al., 2000; Pan et al., 2005; Ji et al., 2021). However, the function of these genes in human TB has not been fully elucidated (Fraschilla and Jeffrey, 2020). Moreover, this mouse model, like other mouse models, is similar to primary and miliary TB, not post-primary TB exhibited by most infected individuals, suggesting that the processes of the development and maintenance of granulomas are different from those in humans and C3HeB/FeJ mice in that respect. High heterogeneity of granulomas has been observed in humans due to the different progression and outcome of the lesions (Lenaerts et al., 2015). Infected lungs in C3HeB/FeJ mice primarily demonstrate three types of granulomatous lesions including necrotic granulomas (Lanoix et al., 2015). However, the necrotic granulomas in all mice examined in this study were pathologically similar. The necrotic granulomas in C3HeB/FeJ mice would exhibit similar progression and development among the same type of lesions, because the initial formation of lesions occurs simultaneously at the time of infection. Since the pathological structure of the necrotic granulomas in C3HeB/FeJ mice resembled those of human encapsulated necrotic granulomas (**Figure 1**), we investigated the components of the necrotic granulomas of C3HeB/FeJ mice in this study.

Recent advances in omics technologies have promoted biomarker research for diseases including infectious diseases (Wheelock et al., 2013; Olivier et al., 2019). Because FM is involved in TB pathogenesis, its characterization will lead to the development of novel TB diagnostic or host-directed therapeutic drugs (Russell et al., 2009; Shim et al., 2020). In the present study, we applied a combination of laser microdissection with proteomic and transcriptomic technologies to identify the potential signatures for FM in *M. tuberculosis*-infected C3HeB/FeJ mouse lungs.

We identified four proteins/genes as the signature candidates for Rim region of necrotic granulomas by spatial multiomic profiling (**Figure 2**). PLIN2 is associated with LD formation and foam cell differentiation in atherosclerosis (Paul et al., 2008). Furthermore, PLIN2 has been demonstrated to localize to FM in human necrotic granulomas (Kim et al., 2010). We found these four signatures localized to Rim region within the necrotic granulomas (**Figure 3**). We also found that PLIN2 and other signatures, MSR1 and LGALS3, but not ARG1, showed a strong correlation with colocalization, whereas proportions of ARG1^+^ PLIN2^-^ cells or ARG1^-^ PLIN2^+^ cells were present (**Figures 4**, **5**), suggesting that MSR1 is one of the markers for FM rather than M2 macrophages in necrotic granulomas. In non-necrotic granulomas, ARG1^+^ cells were distinct from PLIN2^+^ cells (**Figure 6**). These results suggest that ARG1^+^ macrophages migrate to necrotic granulomas and differentiate into FM or that FM are further differentiated to express ARG1, which is in agreement with the results of previous reports that M2 macrophages are associated with FM formation (Mahajan et al., 2012; Oh et al., 2012; Holla et al., 2016). Furthermore, as M2 macrophages possess less bactericidal activities than M1 macrophages against *M. tuberculosis* infection (Huang et al., 2015; Huang et al., 2019), both M2 macrophages and FM derived from M2 macrophages would allow the proliferation of *M. tuberculosis*, leading to FM necrosis and caseum formation.

Since the M2 macrophage markers MSR1 and ARG1 were identified as signatures for FM, we additionally investigated the localization of the markers for M1 macrophages, iNOS and CD68 within granulomas. IHC and IFM analyses suggested that M1 macrophages localized to Rim and Cell regions, as reported previously (Carow et al., 2019), and that M1 macrophage-derived FM were present in necrotic and non-necrotic granulomas (**Figures 3**–**6**). It is possible that *M. tuberculosis* growth is partially restricted in M1 macrophages and M1 macrophage-derived FM, while its restriction is attenuated in the FM of Rim region in necrotic granulomas because of the function of neighboring M2 macrophages or M2 macrophage-derived FM. Therefore, we consider that targeting M2 macrophage-derived FM as well as M2 macrophages within necrotic granulomas is important for developing early detection strategies and host-directed therapies against TB. In the incipient or subclinical TB stages (Drain et al., 2018), development and dissemination of the lesions would follow the formation of necrotic granulomas with the increase of M2-polarized FM. Detection of molecules associated with M2-polarized FM in blood helps an early diagnosis in individuals who develop active TB. For active TB therapy, targeting M2 macrophages and M2-polarized FM within necrotic granulomas using antibodies or chemical compounds would disrupt the proliferative venues for infected *M. tuberculosis* to decrease bacterial burdens and the immuno-suppressive circumstances in infected loci.

Whether LD formation in infected macrophages supports the survival of intracellular pathogens or participates in the host defense mechanism is controversial (Mekonnen et al., 2021). In C3HeB/FeJ mice, *M. tuberculosis* bacilli in Rim of necrotic granulomas displayed more potent proliferation than bacilli in Caseum and Cell (Walter et al., 2021), suggesting that FM in Rim provide a favorable environment for the proliferation of infecting mycobacteria. Multiomic analyses revealed that inflammatory responses in Rim were more enhanced than those in Cell (**Figure 7** and **Supplementary Figure S4**), suggesting that the proliferation of infecting *M. tuberculosis* within FM evokes inflammatory responses. Assuming that LD formation is an essential process for the activation of inflammatory macrophages (Knight et al., 2018; Castoldi et al., 2020), it is possible that infected macrophages are more activated by LD formation, followed by differentiation into FM, while allowing the bacilli’s proliferation.

Neutrophils infiltrate into Caseum of TB lesions and control TB disease progression and pathology (Sarathy and Dartois, 2020). We demonstrated that the activities of inflammatory responses including type I and II IFN responses and TNF signaling were enriched in Caseum by transcriptomics analysis in C3HeB/FeJ mice (**Figure 7**). Proteomic analysis demonstrated that neutrophil markers were enriched in Caseum, while transcriptomic analysis showed partial absence of expression of these genes (**Supplementary Figure S6**), suggesting the necrosis of infiltrated neutrophils. Pathological observation also revealed intact neutrophils in Caseum close to Rim, and acellular caseum and karyorrhectic debris at the center (**Figure 1**) as previously reported (Irwin et al., 2015; Boute et al., 2017). We speculate that infiltration and necrosis of neutrophils in Caseum as well as FM necrosis contribute to the enhancement of inflammatory responses in Caseum.

The expression levels of genes and proteins related to mTORC1 signaling were upregulated in Rim region compared with Caseum or Cell regions by multiomic analyses and qRT–PCR (**Figures 7**, **8**). mTORC1 signaling has been demonstrated to be activated during FM formation in human monocyte-derived macrophages infected with *M. tuberculosis* (Guerrini et al., 2018). Inhibition of mTOR activity has been reported to induce autophagy and to inhibit mycobacterial growth in infected macrophages (Gutierrez et al., 2004). In the context of the concept that the modulation of mTORC1 signaling is a potential target of host-directed therapy in TB treatment (Singh and Subbian, 2018; Guerrini and Gennaro, 2019; Silwal et al., 2021), targeting this pathway in FM will achieve the goal of precision medicine for TB.In conclusion, we identified the signatures for FM in necrotic granulomatous lesions in C3HeB/FeJ mouse lungs and proposed the possibility of heterogeneity in FM activation. The results of this study will provide new insights into the pathogenesis of FM, a niche for proliferation of the pathogen, and regulation of this process may aid the development of novel TB diagnostic and host-directed therapeutic drugs.

## Materials and methods

### Ethics statement

The animal experiments in this study were approved by the Animal Care and Use Committee of The Research Institute of Tuberculosis (RIT) (permit number: No. 2019-01). Animals were treated in accordance with the ethical guidelines of the RIT.

### *M. tuberculosis* culture

*M. tuberculosis* Erdman strain was grown to mid-logarithmic phase in 7H9 medium supplemented with 10% Middlebrook ADC (BD Bioscience), 0.5% glycerol and 0.05% Tween 80 at 37°C as previously described (Seto et al., 2012). Cell suspensions for infection were prepared as previously described (Furuuchi et al., 2022) to remove cell aggregates and clumps (Yamada et al., 2011; Cheng et al., 2014). Briefly, the bacterial culture was filtrated with a 5-μm pore size filter (Pall Corporation) and aliquots of the bacterial solution were stored at -80°C until use. The bacterial number of the stock was determined by CFU assay using 7H10 agar plate medium supplemented with 10% Middlebrook OADC (BD Bioscience) and 0.5% glycerol.

### Mouse model and infection

C3HeB/FeJ mice were purchased from Jackson Laboratory, maintained in a filtrated-air laminar-flow cabinet, and given sterile bedding, water, and mouse chow in the animal facility of the RIT. Specific pathogen-free status was verified by testing sentinel mice housed within the colony. Female mice (6–10 weeks) were transferred into the biosafety level III animal facility of the RIT. Thirteen and 15 mice were infected with *M. tuberculosis via* the intranasal route at a dose of 500 CFU in 30 μL of PBS for multiomic and RT−PCR analyses, respectively. At one day p.i., an average of 183 bacteria (ranging from 100–310 bacteria) were counted in the lungs of six infected mice by CFU assay.

### Sample preparation

At 8 weeks p.i., infected mice were euthanized by cervical dislocation under anesthesia with 0.3 mg/kg of medetomidine, 4.0 mg/kg of midazolam, and 5.0 mg/kg of butorphanol *via* intraperitoneal route (Kawai et al., 2011). The whole lung lobes from infected mice were fixed with 10% formalin neutral buffer solution (Fujifilm Wako Chemicals) for more than 24 hours at 4°C, and were paraffin-embedded. For multiomic analyses, four samples were selected from five infected mice that developed type I necrotic granulomas, according to the number and size of the lesions. For RT-PCR analysis, five mice were selected from six mice that developed type I granulomas. Microdissection was carried out as described in a previous report (Seto et al., 2020). Briefly, mice with isolated necrotic lesions on the lung lobes were selected. The sections with 10-μm thickness from FFPE samples were sliced with a microtome and mounted onto PEN membrane slides (Zeiss). Sections were deparaffinized and stained with hematoxylin, and granulomas were dissected using an LMD700 (Leica). All granulomatous regions were derived from four different mice. One or two necrotic granulomas in the lung lobes of the mouse were microdissected from 10–20 slides and regions containing the areas of 20–50 mm^2^ were collected for the analyses.

### Protein extraction and LC–MS/MS

Microdissected samples were lysed with lysis solvent containing 100 mM HEPES pH 8.0, 10 mM dithiothreitol (DTT), and 0.2% RapiGest SF (Waters) at 95°C for 4 h. The soluble protein solution was separated by centrifugation at 15,000 x g, 4°C for 5 min. For alkylation, iodoacetamide was added to a final concentration of 15 mM and incubated at 37°C for 30 min in the dark. Trypsin (1/50 w/w) was added and incubated at 37°C overnight. Samples were then desalted using a MonoSpin C18 column (GL Science) and dried, followed by redissolution with 0.1% formic acid and filtration with an Ultrafree-MC column (5 µm, Merck Millipore). The samples containing 2 μg of peptides were analyzed by LC–MS/MS as previously reported (Seto et al., 2020).

### RNA extraction, construction of libraries, and sequencing

RNA from microdissected samples was extracted using Quick-RNA FFPE Miniprep (Zymo Research) according to the manufacturer’s instructions. Because of highly degraded RNA in FFPE samples (Von Ahlfen et al., 2007), rRNA depletion of 100 ng RNA from microdissected samples was performed using the NEBNext rRNA Depletion Kit (NEB), followed by the library construction with the NEBNext Ultra II Directional RNA Library Prep Kit (NEB). Libraries were examined with a Qubit fluorometer (Thermo Fisher Scientific) and GenNext NGS Library Quantification Kit (TOYBO) for quantity assessment and DNA High Sensitivity Kit on an Agilent Bioanalyzer 2100 (Agilent) for quality control. Paired-end sequencing (75 bp x 2) of libraries was performed using NextSeq500 (Illumina).

### Bioinformatics

For proteomic analysis, mass spectrometry raw data were processed with MaxQuant (version 1.6.17.0) and searched with the built-in Andromeda search engine (Cox and Mann, 2008) against the UniProt sequence UP000000589 for the identification of mouse proteins. The parameters for the search were set as previously described (Seto et al., 2020). We removed one sample from further analysis because the quality control software, PTXQC (Bielow et al., 2016) showed low quality using the MaxQuant result files (data not shown). Datasets (**Supplementary Table S1**) were analyzed by Perseus software (version 1.6.0.7) (Tyanova et al., 2016). LFQ intensity values were log2-transformed, and missing values were replaced by random numbers drawn from a normal distribution (downshift 1.8, width 0.3). Principal component analysis and hierarchical clustering were performed on these datasets. Differentially expressed proteins were identified using the cutoff of Welch’s *t*-test with false discovery rates (FDR) of 0.05 and 0.2 for comparisons between two regions and between Rim and the other two regions, respectively. Gene ontology biological processes (GOBP) enrichment analysis for the differentially expressed proteins was performed with DAVID (Huang da et al., 2009) and Metascape (Zhou et al., 2019).

For transcriptomic analysis, raw reads were processed with Trim Galore (version 0.6.6) for read-quality trimming (https://github.com/FelixKrueger/TrimGalore). The processed reads were then aligned with HISAT2 (version 2.2.1) (Kim et al., 2019) against mouse genome mm10. Gene counts were determined with featureCounts (version 2.0.1) (Liao et al., 2014). We removed one sample from further analysis, because more than 98% of its reads corresponded to sequences other than mRNA by Picard Toolkit (https://github.com/broadinstitute/picard) (**Supplementary Table S4**). Normalization and differential expressed gene analysis were carried out with edgeR (version 3.28.1) (Robinson et al., 2010) using generalized linear models and quasi-likelihood tests (Lun et al., 2016) to generate datasets (**Supplementary Table S2**). GSEA was performed based on transcript per million (TPM) data with hallmark gene sets collected from the Molecular Signature Database (MSigDB) (Subramanian et al., 2005). Significantly enriched gene sets were determined using the cutoff of the adjusted *P* value at 0.25.

Heatmap, hierarchical clustering analysis, Venn diagram analysis, ANOVA, Dunn’s multiple comparison test, and Dunnett’s multiple comparison test were performed using R version 3.6.3.

### Data availability

Mass spectrometry raw files have been deposited in the ProteomeXchange consortium *via* the jPOST partner repository with the dataset identifier JPST001401/PXD030088 (Okuda et al., 2017). Raw sequence data have been deposited in the DRA database under the accession number DRA013139.

### IHC and IFM

IHC and IFM were carried out as previously described (Seto et al., 2020) except for the antigen retrieval process; deparaffinized and rehydrated samples on slides were treated with a solution containing 1% ImmunoSaver (Nisshin EM) at 98°C for 45 min. Antibodies used in this study are listed in **Supplementary Table S5**. Samples for IFM were observed with an Olympus IX81 microscope equipped with a DP74 camera (Olympus). Images were processed and analyzed with Fiji software (Schindelin et al., 2012). Ziehl-Neelsen (ZN) staining and Elastica van Gieson (EVG) staining were carried out according to the manufacturer’s instructions (Muto Pure Chemical). Quantitative analysis of the colocalization of the indicated marker proteins with PLIN2 was performed as shown in **Supplementary Figure S7**. Briefly, the images for PLIN2 and the indicated protein were converted to binary images. The inverted image of the indicated protein was subtracted from the image of PLIN2 to obtain the image showing the area for the merged image between PLIN2 and the indicated protein. The colocalization index was calculated by dividing the area of the merged image with that of PLIN2.

## Data availability statement

The datasets presented in this study can be found in online repositories. The names of the repository/repositories and accession number(s) can be found below: https://repository.jpostdb.org/entry/JPST001401, https://ddbj.nig.ac.jp/resource/sra-submission/DRA013139.

## Ethics statement

The animal study was reviewed and approved by The Animal Care and Use Committee of The Research Institute of Tuberculosis.

## Author contributions

SS, MH, and NK designed the project. SS, HN, and HH performed animal experiments. SS and TN performed proteomic analysis. SS, TCG, KW, AM, and MH performed transcriptomic analysis. SS, TN, MH, and NK wrote and revised the manuscript. All authors approved the manuscript.

## Funding

This study was supported by the Emerging/Re-emerging Infectious Diseases Project of the Japan Agency for Medical Research and Development (21fk0108090, 21fk0108127, 21fk0108607, 21wm0225011), and Grants-in-Aid for Scientific Research, Japan Society for the Promotion of Science (19K07552, 22K07065).

## Acknowledgments

We thank Mr. Toshiaki Aoki, Ms. Miyako Seto, and Ms. Yuki Inagaki in Department of Pathophysiology and Host Defense, the RIT for preparation of FFPE slides, IHC, and animal experiments, Dr. Hiroshi Kido in Institute of Advanced Medical Science, Tokushima University for technical consulting about intranasal infection, Dr. Takuya Kitamoto, Dr. Minako Kondo, and Mr. Yuki Kurita in Department of Advanced Research Facilities and Services, Hamamatsu University School of Medicine for the operation of LC–MS/MS and the staining of FFPE slides, and Dr. Takashi Minowa in Nanotechnology Innovation Station, National Institute of Materials Science (NIMS) for the operation of LMD700. This study was supported by NIMS Molecule & Material Synthesis Platform in “Nanotechnology Platform Project” operated by the Ministry of Education, Culture, Sports, Science and Technology, Japan.

## Conflict of interest

The authors declare that the research was conducted in the absence of any commercial or financial relationships that could be construed as a potential conflict of interest.

## Publisher’s note

All claims expressed in this article are solely those of the authors and do not necessarily represent those of their affiliated organizations, or those of the publisher, the editors and the reviewers. Any product that may be evaluated in this article, or claim that may be made by its manufacturer, is not guaranteed or endorsed by the publisher.

## Supplementary material

Supplementary Figure 1 Heatmap of hierarchical clustering of genes with upregulated expression in Rim regions by proteomics and transcriptomics. Hierarchical clustering analyses of differentially expressed proteins and genes were carried out based on LFQ values **(A)** or transcripts per million (TPM) **(B)** for proteomic and transcriptomic analyses, respectively. Candidate signatures are underlined.

Supplementary Figure 2 (A) Heatmap of *Msr1*, *Lgals3*, and *Plin2* in the indicated immune cells using RNA-seq data from ImmGen (Heng and Painter, 2008). The relative value of gene expression is shown. (B) The proportion of PLIN2^+^ cells in CD68^+^, CD163^+^, or CD206^+^ macrophages. The averages and standard deviations of PLIN2^+^ cells in macrophages are shown. More than 50 images of IFM for indicated marker proteins and PLIN2 were analyzed.

Supplementary Figure 3 Localization of M2 macrophage markers, CD163 and CD206 to Rim of necrotic granulomas. **(A, B)** IHC for the M2 macrophage markers CD163 **(A)** and CD206 **(B)** in necrotic granulomas. **(C, D)** IFM for CD163 **(C)** and CD206 **(D)** to PLIN2^+^ cells. Arrows and arrowheads indicate both marker and PLIN2-positive cells, and marker-negative, PLIN2-positive cells, respectively.

Supplementary Figure 4 Profiling of protein expression in granulomatous regions. Gene ontology (GO) terms related to the biological process of the differentially expressed proteins were examined. Heatmaps represent the top 20 ranked enriched GO terms clustered in comparing significantly abundant proteins between Caseum and Cell **(A)**, Rim and Caseum **(B)**, or Rim and Cell **(C)** regions. The color bar indicates a negative logarithmic adjusted *P* value (-log (p.adj)). Between Caseum and Cell, proteins related to phagocytosis and immune cell migration were found in Caseum region, while proteins related to antigen presentation and IFN-γ response were found in Cell region. Proteins related to phagocytosis are expressed in neutrophils, and proteins related to antigen presentation and IFN-γ response, such as MHC class II molecules and proteasomes, are expressed in antigen presenting cells. Between Rim and Caseum regions, proteins related to cell redox homeostasis, protein folding and response to interleukin-7 were enhanced in Rim region, and those of response to oxidative stress and lipoprotein modification were enriched in Caseum region. Between Rim and Cell regions, proteins related to inflammation and phagocytosis were enriched in Rim region, while cellular metabolic pathways were enriched in Cell region. Collectively, the expression levels of proteins related to phagocytic activity and immune cell migration were increased from Cell region to Caseum region, and conversely, those of antigen presentation and cell metabolism were higher from Caseum region to Cell region.

Supplementary Figure 5 Heatmaps showing the expression of core enriched genes related to fatty acid metabolism, oxidative phosphorylation and mTOR signaling for GSEA.

Supplementary Figure 6 Heatmap showing the expression of neutrophil marker proteins **(A)** and genes **(B)** in necrotic granulomas.

Supplementary Figure 7 Illustration for calculating the colocalization index in Figure 5.

Supplementary Table 1 Excel file of the raw and imputed data of all proteomic analyses for the identification of proteins of necrotic granulomatous lesions.

Supplementary Table 2 Excel file of transcriptomic data analyzed with edgeR.

Supplementary Table 3 The core enrichment genes related to fatty acid metabolism, oxidative phosphorylation and mTOR signaling for GSEA.

Supplementary Table 4 RNA-seq metrics using Picard CollectRnaSeqMetrics. PF, passing filter.

Supplementary Table 5 Antibodies used in this study.
