# Supplementary figures and images for: Spatial multiomic profiling reveals the novel polarization of foamy macrophages within necrotic granulomatous lesions developed in lungs of C3HeB/FeJ mice infected with Mycobacterium tuberculosis
Source: Front Cell Infect Microbiol. 2022 Sep 27;12:968543. doi: 10.3389/fcimb.2022.968543 (PMC9551193; doi:10.3389/fcimb.2022.968543)

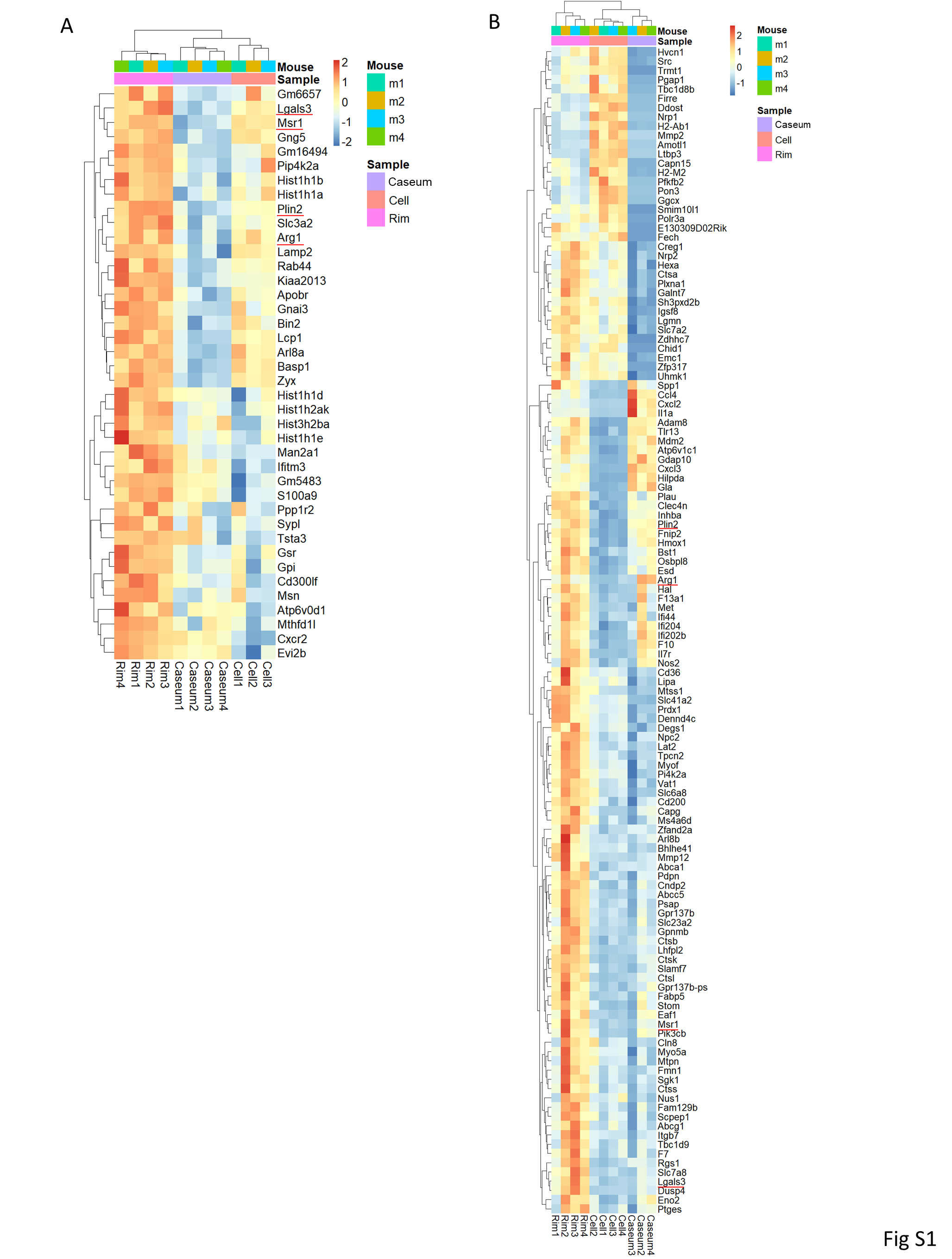

Supplement: Supplementary Figure 1 — Heatmap of hierarchical clustering of genes with upregulated expression in Rim regions by proteomics and transcriptomics. Hierarchical clustering analyses of differentially expressed proteins and genes were carried out based on LFQ values (A) or transcripts per million (TPM) (B) for proteomic and transcriptomic analyses, respectively. Candidate signatures are underlined. [file Image_1.tif]

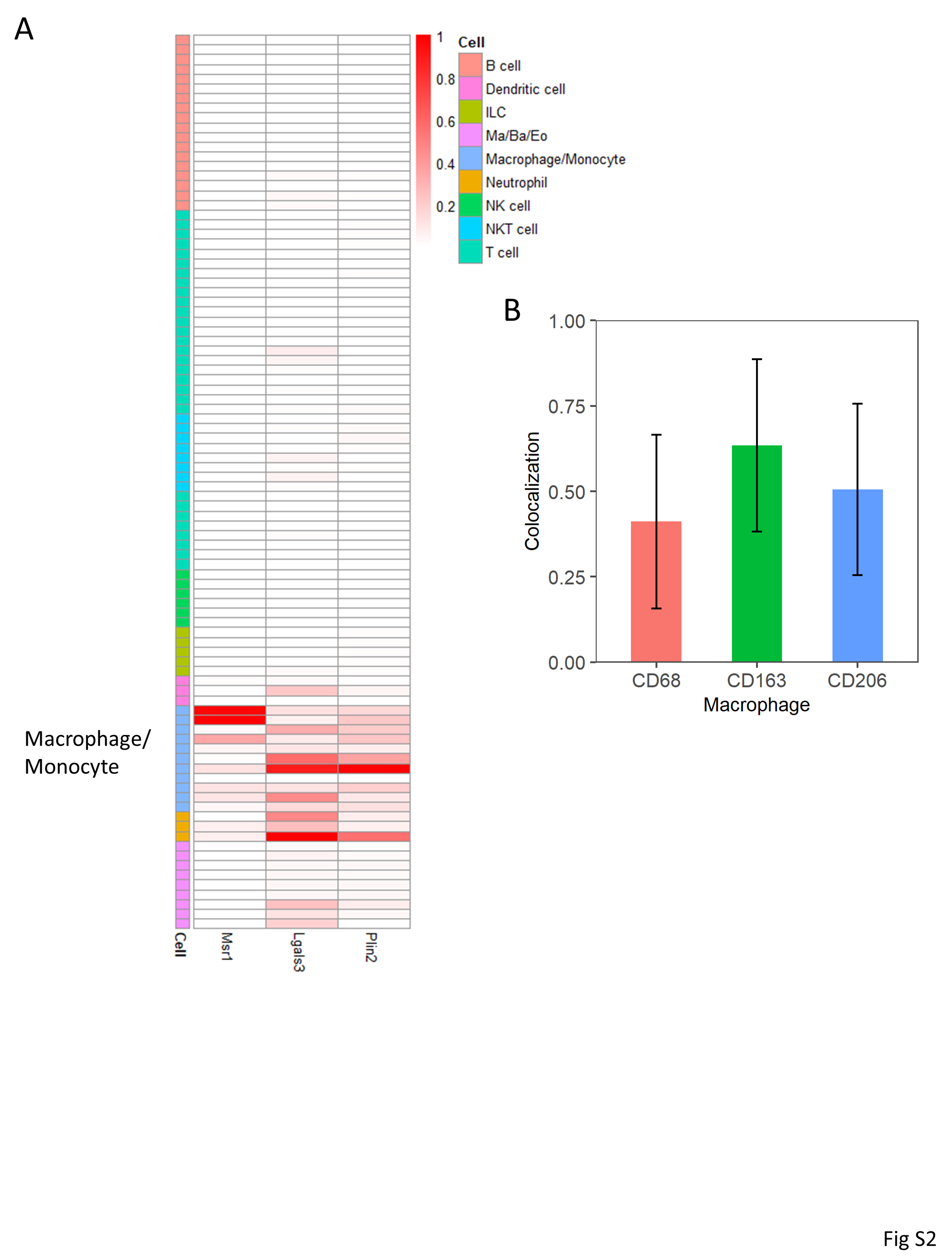

Supplement: Supplementary Figure 2 — (A) Heatmap of Msr1, Lgals3, and Plin2 in the indicated immune cells using RNA-seq data from ImmGen (Heng and Painter, 2008). The relative value of gene expression is shown. (B) The proportion of PLIN2+ cells in CD68+, CD163+, or CD206+ macrophages. The averages and standard deviations of PLIN2+ cells in macrophages are shown. More than 50 images of IFM for indicated marker proteins and PLIN2 were analyzed. [file Image_2.tif]

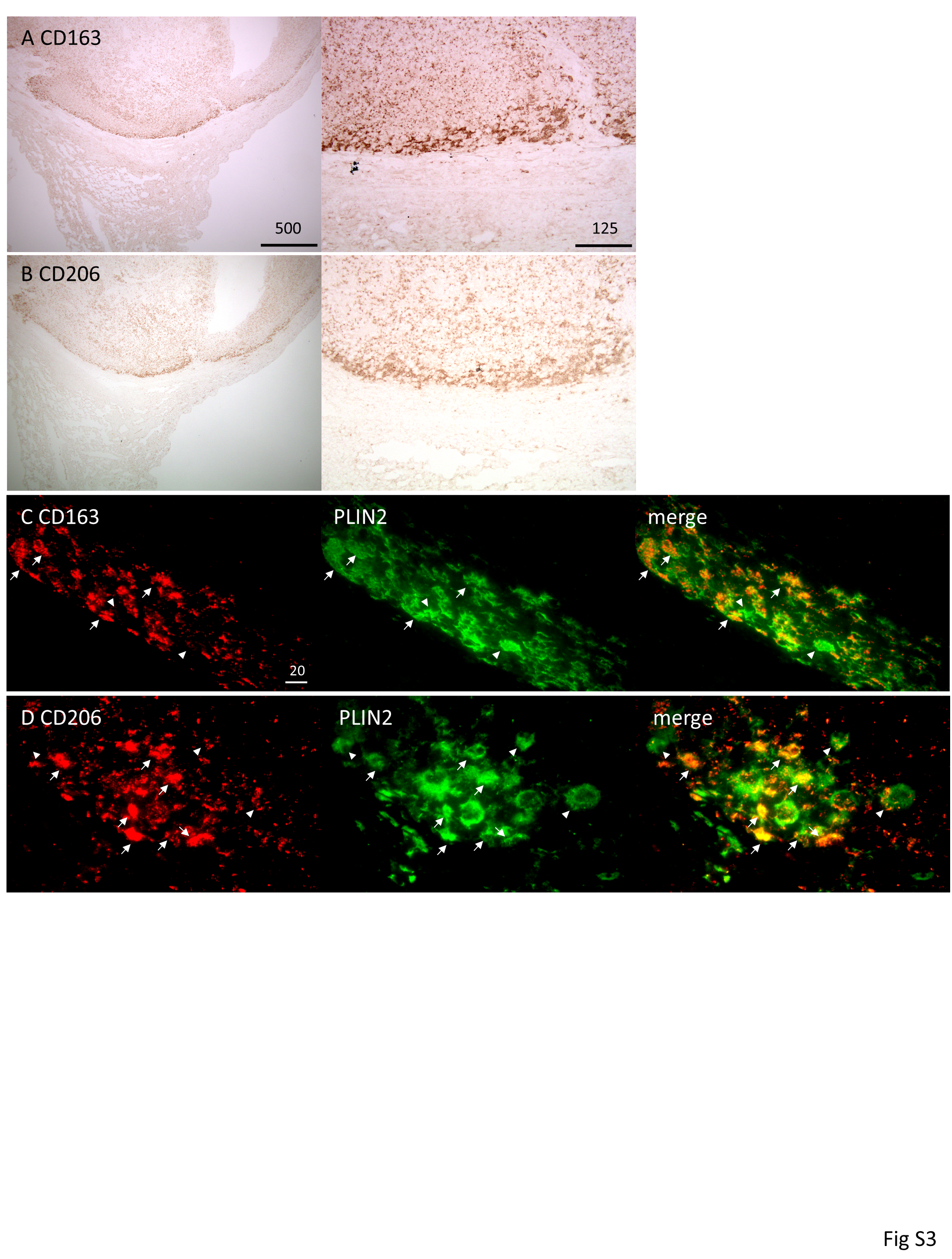

Supplement: Supplementary Figure 3 — Localization of M2 macrophage markers, CD163 and CD206 to Rim of necrotic granulomas. (A, B) IHC for the M2 macrophage markers CD163 (A) and CD206 (B) in necrotic granulomas. (C, D) IFM for CD163 (C) and CD206 (D) to PLIN2+ cells. Arrows and arrowheads indicate both marker and PLIN2-positive cells, and marker-negative, PLIN2-positive cells, respectively. [file Image_3.tif]

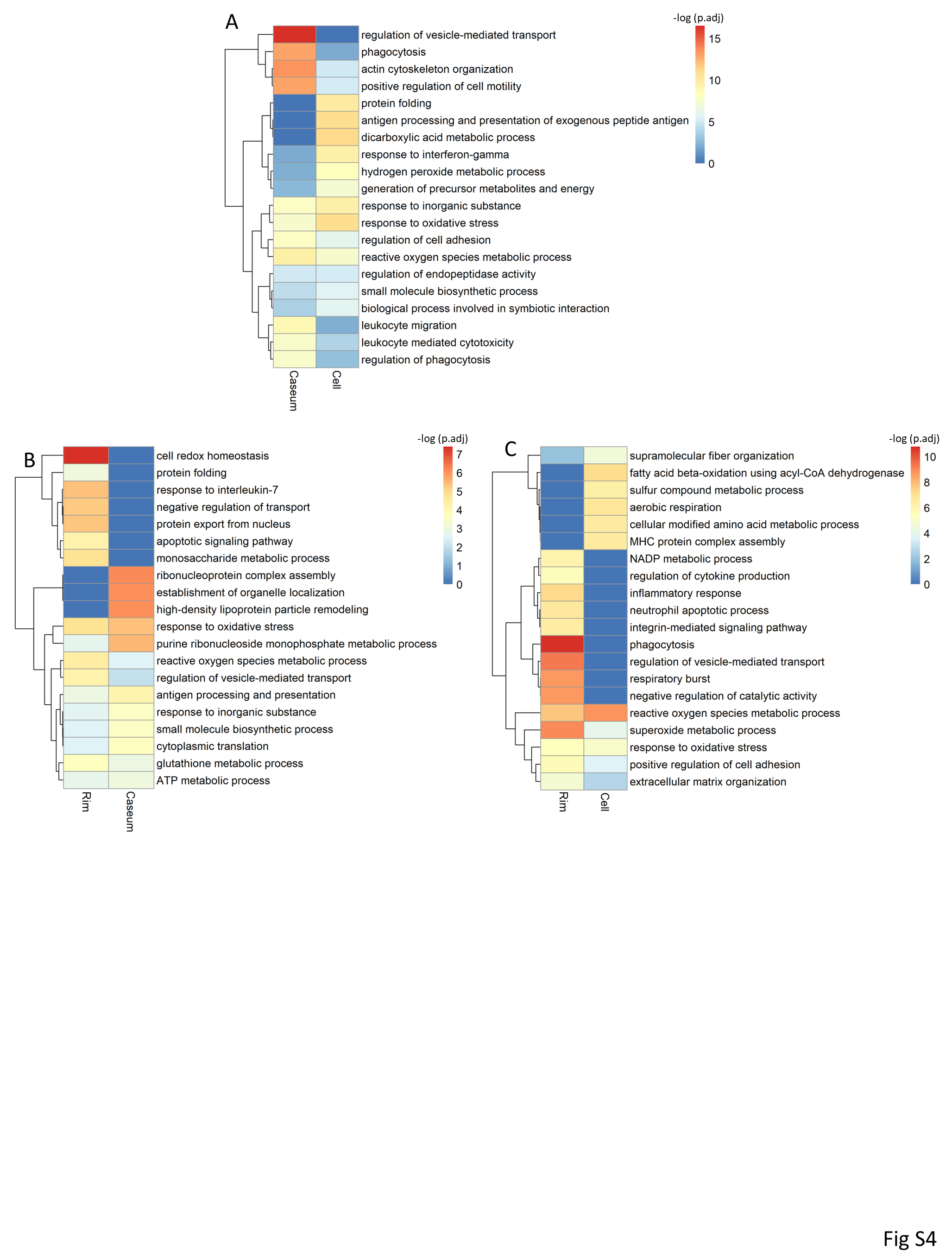

Supplement: Supplementary Figure 4 — Profiling of protein expression in granulomatous regions. Gene ontology (GO) terms related to the biological process of the differentially expressed proteins were examined. Heatmaps represent the top 20 ranked enriched GO terms clustered in comparing significantly abundant proteins between Caseum and Cell (A), Rim and Caseum (B), or Rim and Cell (C) regions. The color bar indicates a negative logarithmic adjusted P value (-log (p.adj)). Between Caseum and Cell, proteins related to phagocytosis and immune cell migration were found in Caseum region, while proteins related to antigen presentation and IFN-γ response were found in Cell region. Proteins related to phagocytosis are expressed in neutrophils, and proteins related to antigen presentation and IFN-γ response, such as MHC class II molecules and proteasomes, are expressed in antigen presenting cells. Between Rim and Caseum regions, proteins related to cell redox homeostasis, protein folding and response to interleukin-7 were enhanced in Rim region, and those of response to oxidative stress and lipoprotein modification were enriched in Caseum region. Between Rim and Cell regions, proteins related to inflammation and phagocytosis were enriched in Rim region, while cellular metabolic pathways were enriched in Cell region. Collectively, the expression levels of proteins related to phagocytic activity and immune cell migration were increased from Cell region to Caseum region, and conversely, those of antigen presentation and cell metabolism were higher from Caseum region to Cell region. [file Image_4.tif]

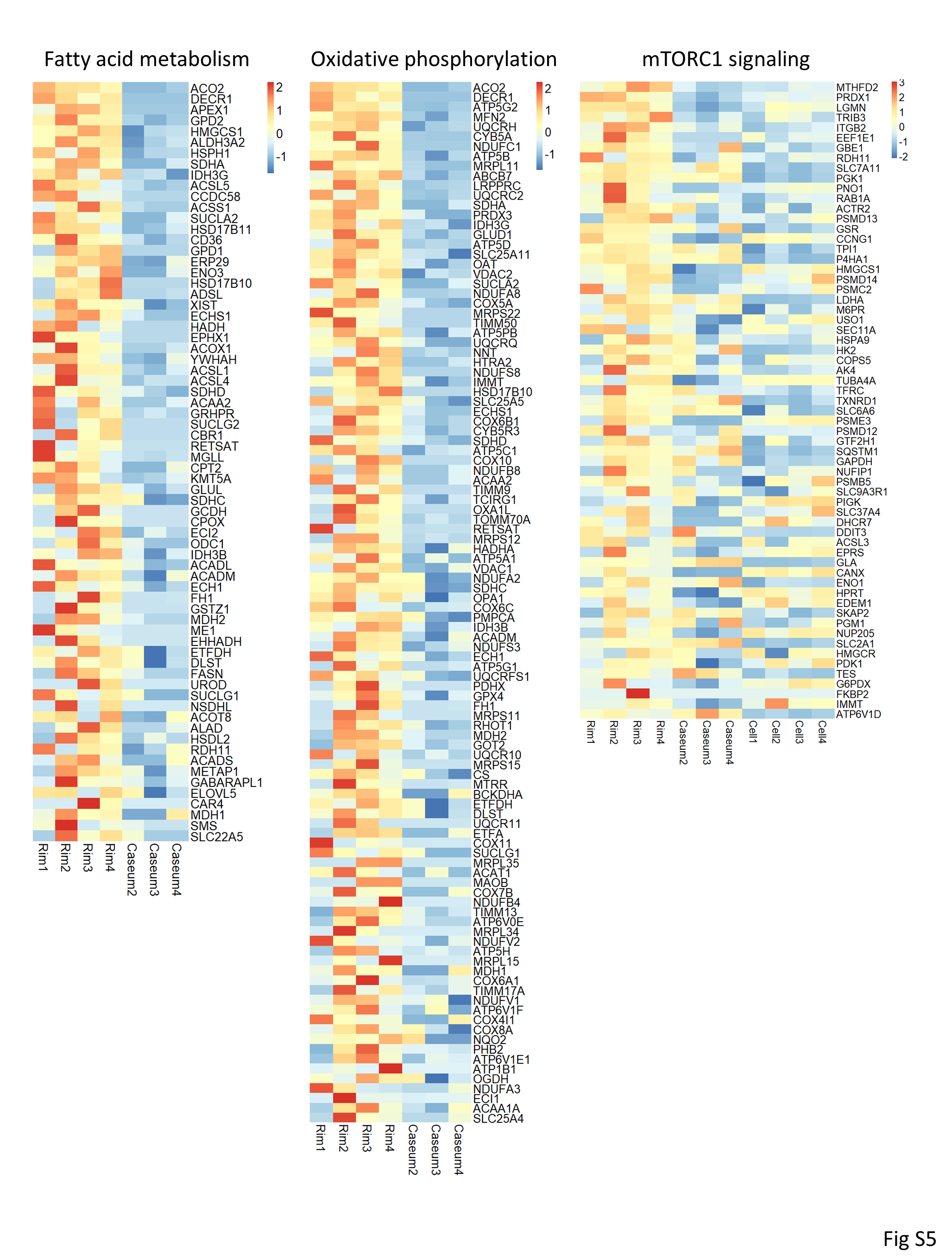

Supplement: Supplementary Figure 5 — Heatmaps showing the expression of core enriched genes related to fatty acid metabolism, oxidative phosphorylation and mTOR signaling for GSEA. [file Image_5.tif]

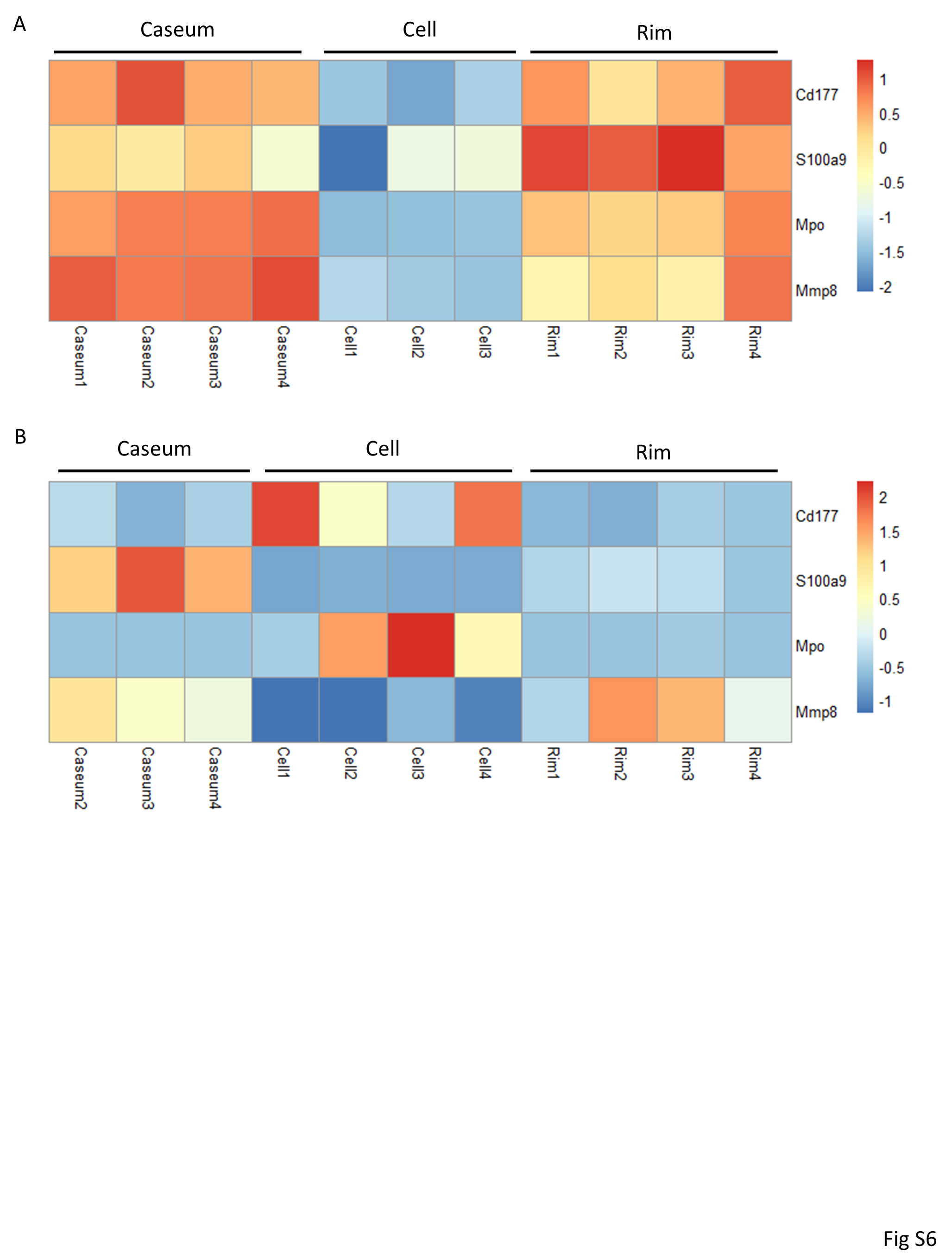

Supplement: Supplementary Figure 6 — Heatmap showing the expression of neutrophil marker proteins (A) and genes (B) in necrotic granulomas. [file Image_6.tif]

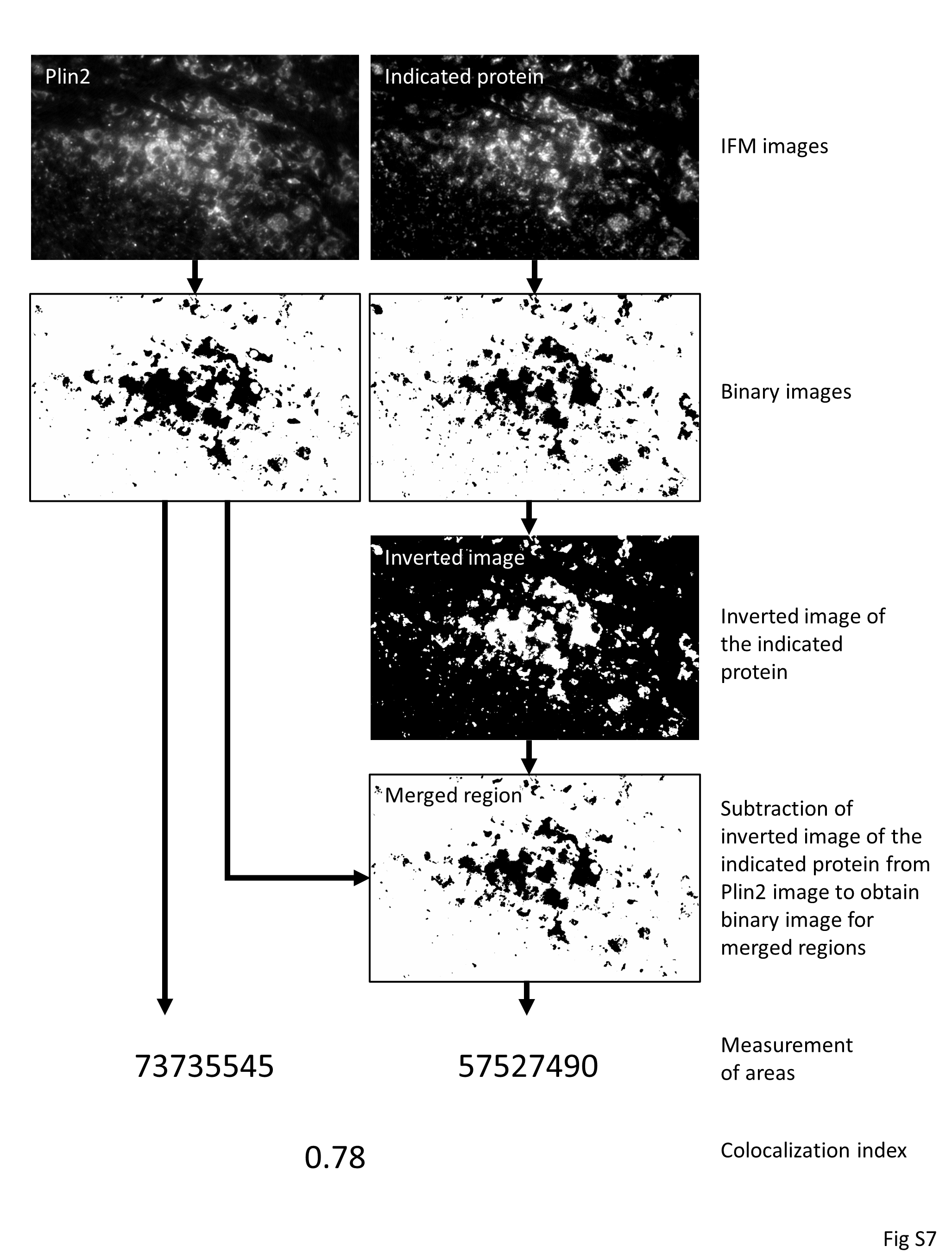

Supplement: Supplementary Figure 7 — Illustration for calculating the colocalization index in Figure 5. [file Image_7.tif]
